# Role of Organic and Eco-Friendly Inhibitors on the Corrosion Mitigation of Steel in Acidic Environments—A State-of-Art Review

**DOI:** 10.3390/molecules26113473

**Published:** 2021-06-07

**Authors:** Hyun-Min Yang

**Affiliations:** 1 Department of Chemical and Environmental Engineering, University of California-Riverside, Riverside, CA 92521, USA; yhm_khan@naver.com; 2 Innovative Durable Building & Infrastructure Research Center, Department of Architectural Engineering, Hanyang University, Ansan-si 15588, Korea

**Keywords:** steel, acid, corrosion, eco-friendly inhibitor, efficiency, pickling, descaling, boiler tube

## Abstract

Steel has versatile application in chemical, structure and construction industries owing to its mechanical properties. However, it is susceptible to corrosion in acid environments. Thus, it requires to protect the steel from corrosion. Different types of corrosion resistance steel, coatings and inhibitors are developed to mitigate the corrosion, but, inhibitor is the best remedies to control the corrosion of steel in acid condition. Moreover, organic and green inhibitors used in acid condition for descaling, acid pickling, pipelines, boiler tubes and oil-wells. Organic inhibitors reduce the dissolution of steel in acid but, it is hazardous, expensive and needs expertise to synthesize the inhibitor. Therefore, there is utmost required to study and compile the latest research about the eco-friendly corrosion inhibitors, which showed more than 90% corrosion inhibition efficiency. In the present study, I have reviewed the state-of-arts, and compile the latest development in organic and eco-friendly corrosion inhibitor used in acid environment as well as suggested about the future scope and role of green inhibitor for sustainable society, which is economical, less hazardous and readily available from the natural sources.

## 1. Introduction

Steel is considered as a versatile material for fabrication of structures, equipment and various components of home appliances and industrial applications [1]. Steels have numerous applications such as construction of bridges, buildings, railway tracks, engines, furniture, refrigerators and washing. The inherent tendency of steels is to interact with their surroundings and develop unstable and non-protective reaction products at the surface [2]. However, it makes them unsuitable for use in many applications. Tremendous efforts have been made and are still being attempted all over the world to make the steels immune or passive towards its surroundings. These efforts include to develop alloys; provide electrochemical protections and suitable inhibitors to corrosive environments, new types of paints and metallic and non-metallic surface coatings.

To achieve a greater ratio of load carrying capacity to weight of the steel and hence to increase fuel efficiency, special types of micro alloyed steel and high strength steel sheets are now being used in transportation and building industries. A study conducted by the American Iron and Steel Institute (AISI) demonstrate that careful design and utilization of high strength steels could reduce the mass of a midsized steel auto body by 30% without having any adverse effect on performance and cost [3]. Due to heavy cold working and reduced thickness, these sheets, however, become extremely prone to perforation types of corrosion attack. Fortunately, during the last 50 years, the understandings about corrosion science and engineering have advanced to a great extent, which has helped modern age industries, tremendously, in controlling the corrosion. There are different types of rust/scales formed on the steel substrates, which can be removed by different types of organic and inorganic acids such as HCl, H_2_SO_4_, phosphoric acid, acetic acid and formic acid. Choice of acid depends on solubility of oxide/scale in acid solution and composition of various components in steel. In the descaling process, the metal loss is higher than experienced in actual atmospheric exposure. Following reactions are helpful to elucidate the nature of steel corrosion:

The first step of steel corrosion is as follow [4]:

Fe → Fe^2+^ + 2e^−^ (1)

Equation (1) is considered as an anodic reaction. When steel corrodes, the rate is controlled by cathodic reaction. Some cathodic reactions are as follows:

Hydrogen evolution  2H^+^ + 2e^−^ → H_2_ (2)

Oxygen reduction (in acidic solution) O_2_ + 4H+ + 4e^−^ → 2H_2_O (3)

Oxygen reduction (basic or neutral solution) O_2_ + 4H_2_O + 4e^−^ → 4OH^−^ (4)

Hydrogen evolution and oxygen reduction are very common reactions in acid medium. The cathodic or anodic reactions can be reduced by inhibitors.

### 1.1. Corrosion Reaction Mechanism of Steel in Acid Solution

Acid solutions i.e., HCl and H_2_SO_4_ are used in chemical laboratories and industries for cleaning, pickling, de-scaling, oil-well cleaning and pipeline cleaning [5]. The corrosion mechanism of iron in aqueous HCl solution can be illustrated as [6,7]:

(5) $$\mathrm{Fe}+{\mathrm{Cl}}^{-}\;⇋\;{\mathrm{FeCl}}^{-}{\;}_{\mathrm{ads}}$$

(6) $${\mathrm{FeCl}}^{-}{\;}_{\mathrm{ads}}+\mathrm{Fe}\;⇋\;{\mathrm{FeCl}}^{-}{\;}_{\mathrm{ads}}\mathrm{Fe}$$

FeCl^−^ _ads_Fe + OH^−^ → FeOH^+^ + Fe + Cl^−^ + 2e^−^ (7)

(8) $${\mathrm{FeOH}}^{+}+H^{+}\;⇋\;{\mathrm{Fe}}^{2+}\;+\;H_{2}O$$

### 1.2. Corrosion Mitigation of Steel in the Presence of Inhibitor

**Figure 1:**
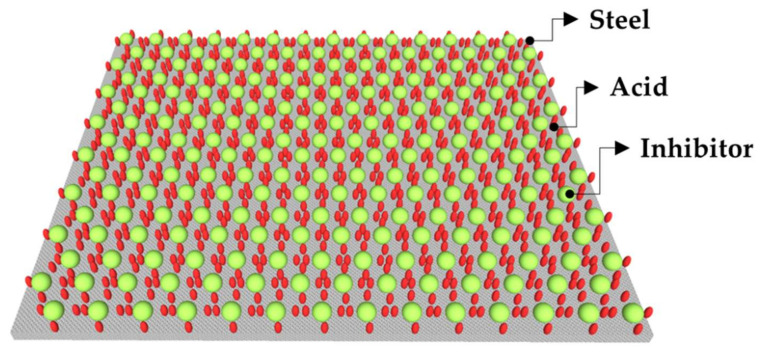
Adsorption action of inhibitor on steel surface.

To cease the effect of acid solution on steel surface, inhibitors are added to the acid solutions at moderate temperature. Inhibitors must be stable and effective in hot concentrated acid even in severe environment [8]. It is not necessary that a particular inhibitor would be effective in every acid solution, therefore, it is suggested that there has to be one specific inhibitor for a specific solution [9]. The choice of inhibitor depends on type of acid, its concentration, temperature and velocity of flow, the presence of dissolved inorganic and organic substances and types of steel exposed in the acidic solution. Inhibitor should also be thermally and chemically stable; it should have good surfactant and foaming characteristics [10]. Organic compounds are considered to be the most effective and efficient inhibitors because they have hetero atoms, i.e., O, N and S, as well as π (pi) bonds which, adsorbed onto the steel substrate as shown in Figure 1. The hetero atoms donate lone electrons to steel (Fe) and form very thin and persistent adsorbed film that lead to reduce the corrosion owing to the slow down the anodic, cathodic and both reactions. They form very strong covalent bond and adsorbed onto the steel substrate (Figure 1). There are different types of adsorption phenomena of inhibitor molecules i.e., chemisorption, physio-sorption and physiochemical adsorption. Inorganic compounds are also used as inhibitors such as chromate, dichromate and nitrite [11,12,13,14,15,16]. However, the uses of chromate are already banned for years due to the several negative effects [17]. Thus, it is recommended to develop novel corrosion inhibitors from natural source and non-toxic, which put negligible negative impact in environment [18]. There are various types of organic and inorganic inhibitors, which tend to reduce the corrosion rate of steel and iron in acid solution [19,20,21,22]. Mainly HCl is used in industries and laboratories for pickling, cleaning and de-scaling of steel and ferrous alloys [23].

There is different area for the application of inhibitor in acid condition. In the subsequent paragraphs, one-by-one will be discussed.

## 2. Inhibitor Used during Acid Pickling of Steel

Pickling inhibitors are interfacially active organic or inorganic substances. As a result of electrostatic interactions, they cover the iron surface with a thin passive layer and reduce acid attack. The interaction depends on polarity of metal surface and composition of inhibitor and pickling medium [24]. Different types of inhibitors are used as pickling solutions i.e., organic and inorganic inhibitors. The use of inorganic inhibitors as an alternative to organic compound is based on possibility of degradation of organic compounds with time and temperature [25]. Mostly organic inhibitors are employed as acid inhibitors; as the passivating inorganic inhibitors may be dangerous in acid environments and cause severe localized attack once the passive film gets broken [26]. Choudhary et al. [27] proposed that cetyl trimethyl ammonium chloride (CTMAC) and cetyl trimethyl ammonium bromide (CTMAB) inhibits corrosion of mild steel in 1 M HCl solution, in which, CTMAC is more efficient than CTMAB. Zhang et al. [28] investigated that 1-butyl-3-methylimidazolium chloride (BMIC) and 1-butyl-3-methylimidazolium hydrogen sulfate [(BMIM)HSO4] inhibit corrosion of mild steel in 1 M HCl. The inhibition efficiency was more effective with inhibitor concentration. Between these two inhibitors, alkylimidazolium base inhibitor [(BMIM)HSO_4_] is more efficient than BMIC. Ashassi et al. [29] investigated that Prunus juice acts as a green inhibitor for corrosion of steel in HCl media and they have found that its efficiency is increased as the concentration increased, but it decreased by increasing temperature. Shukla et al. [30] studied inhibitory effect of Cefadroxil on mild steel in 1 M HCl. The inhibitor showed 96% efficiency at 11.0 × 10^−4^ mol L^−1^ concentration. Ashassi-Sorkhabi et al. [31] proposed that three amino acids, glycine, alanine and leucine, acts as a green inhibitor for steel in HCl solution. It acts as an inhibitor through adsorption on steel surface. Its inhibitory effects range from 28–91%. The efficiency depends on type of amino acid and concentration. Kumar et al. [32] investigated cloxacillin antibiotic as a corrosion inhibitor. It is eco-friendly and a good inhibitor for mild steel in 1 M HCl. It showed 81% efficiency at 15 × 10^−4^ M. Bentiss et al. [21] concluded that 2,5,-disubstituted-1,3,4-oxadiazole is a good inhibitor for mild steel in 1 M HCl. It acts on the cathodic reaction without changing the mechanism of H_2_ evolution reaction. It adsorbs on metal surface leading to formation of passive film, which grows with time and protect steel from attack of acid solution. Shukla et al. [33] showed that five triazoles namely Hexahydro-1,3,5-triphenyl-s-triazine (Inh-1), Hexahydro-1,3,5-p-tolyl-s-triazine (Inh-2), Hexahydro-1,3,5-p-methoxyphenyl-s-triazine (Inh-3), Hexahydro-1,3,5-p-aminophenyl-s-triazine (Inh-4) and Hexahydro-1,3,5-p-nitrophenyl-s-triazine (Inh-5) were synthesized and investigated as inhibitors for mild steel in 1N HCl. The order of efficiency of inhibitor was Inh-4 > Inh-3 > Inh-2 > Inh-1 > Inh-5. All triazines showed inhibitor efficiency at 300 ppm concentration. Presence of –NH_2_ group in inh-4 increased its efficiency while –NO_2_ reduced the efficiency of inh-5. Elewady [34] investigated that 2,6-Dimethylpyrimidine-2-amino and its two derivatives, i.e., N-Benzilidene-4,6, dimethyl pyrimidine-2-amine and 2-[(3,6-Dimethylpyrimidine-ylimino) methyl]-4-nitrophenol, have been investigated as corrosion inhibitors for C-steel in 2 M HCl. N-Benzilidene-4,6, dimethyl pyrimidine-2-amine gives the highest inhibitor efficiency due to its adsorption mechanism. Obi-Egbedi et al. [35] studied 1,2-diaminoanthraquinone as a corrosion inhibitor in 1 M HCl on mild steel at temperature (303–333 K). Its efficiency increases with increase in concentration but decrease with rise in temperature. Zhang et al. [36] have synthesized imidazoline-based dissymmetric bis-quaternary ammonium gemini surfactant, which acts as a good corrosion inhibitor for Q 235 steel in 1 M HCl. They adsorb on iron surface firmly by donating its pi-electrons to Fe and accepting electrons from d-orbitals of Fe. Ahmad et al. [37] investigated the inhibitory effect of Albedazole on mild steel in 1 M HCl. It has a benzimidazole ring with delocalized pi-electrons and a methyl carbamate group (-NHCOOCH_3_). This structure favors the interaction of Albedazole with metal. Maghraby and Soror [38] investigated the effect of cetyl trimethyl ammonium bromide as cationic surfactant on the corrosion of C-steel in HCl solution. Its effect was tested in 1 M and 2 M HCl solution and found that it gives best result in 2 M HCl solution. The inhibition efficiency value increases with inhibitor dose and decreases with the temperature.

Inhibitor Used to Control the Corrosion in Pipeline

Pipelines are the safest mode for transporting large quantities of crude oil and natural gas over land, because large scale transportation of crude oil and natural gas by tanker trucks and trains are not possible. Pipelines are the alternative mode of transportation. Underground pipelines are safer mode for transportation. Liquid pipelines are used for transportation of liquid or natural gas liquids such as ethane, propane, butane and natural gas pipelines from oil wells. It is gaseous hydrocarbon contains methane lesser amount of CO_2_, H_2_S, N_2_, H_2_O and organic acids. These are refining and transported. Oil and gas pipelines are made up of carbon steel. Oil is hydrophobic in nature, therefore, it possesses no harmful effect on pipeline walls, but some moisture content, gases, CO_2_ and H_2_S passes through pipelines which are highly corrosive. Therefore, corrosion inhibitor is used to protect oil and gas pipelines. However, an inhibitor should be present in the water phase so that it cannot reach the pipe walls [39]. Corrosion occurs on water wetted surface, so the inhibitor should contain active components to provide protection. Corrosion also arises due to presence of solid deposits sand, scale and corrosion products. It constructs an extra layer that the corrosion inhibitor has to overcome [39]. It also adsorbs active components of the inhibitor [40,41,42] and promotes growth of bacteria, which causes microbial corrosion. Therefore, an adequate corrosion inhibitor is required for protection [43]. Two types of corrosion inhibitors are required according to the type of aggressive media (i) water soluble and (ii) hydrocarbon soluble. Water soluble inhibitors are used in transportation and production of oil and scale removal process [44]. Corrosion caused by CO_2_ (sweet corrosion) and H_2_S (sour corrosion) are most prevalent form of attack found in oil and gas production [45]. HCl is formed as by-product in refineries. H_2_S concentration and solution pH can either accelerate or slow down the corrosion of iron. In small concentrations, i.e., 40 µmol cm^−3^, H_2_S forms an FeS protective layer. Abelev et al. [46] examined that a small H_2_S concentration (5 ppm) have an inhibiting effect on corrosion in presence of CO_2_ at temperature between 25–55 °C. Nitrogen based inhibitors such as imidazolines and their salts have been used in oil and gas production system to control internal corrosion of carbon steel structure [47,48].

E.F. Diaz [49] evaluated H_2_S corrosion inhibition of X-120 pipeline steel by using carboxyethylimidazoline by electrochemical techniques. The effect of carboxyethylimidazolin concentration on corrosion inhibition of X-120 steel in 3% NaCl and at 50 °C temperature has been studied and it is found that the best corrosion inhibition is found by adding 160 µmol^−1^ carboxyethylimidazolin. Zagidullin et al. [50] proposed a teraphthalic acid based method for corrosion inhibition. An acid inhibitor is produced by interaction of polyethylene-polyamide with teraphthalic acid at 150–190 °C for 48 h in the molar ratio of 2:1, followed by reaction with benzyl chloride at 80 °C for 5 h. The product formed was used with urotropin and neonol in water. Reznik [51] patented Rhodanine (2-thioxo-4-thiazolidinone) and its five derivatives as Fe corrosion inhibitor for oil refining equipment in carbonic acid. Subramaniyam et al. [52] patented a polyisobutylene phosphorous sulfur compound as corrosion inhibitor for nephthenic acid corrosion and sulfur corrosion. Alykov et al. [53] patented 1-nitro-3,3-diphenyl-1-[3-(3-nitrophenyl)-1,2,4-oxydiazol-5-yl]-2,3-diazoprop-1-ene obtained by condensation reaction of equimolar quantities of substituted 3-aryl-5-nitromethyl-1,2,4-oxadiazol with 1,1-diphenylhydrazine in ethoxyethane medium as a corrosion inhibitor for metal protection against acid corrosion in oil and gas pipeline for non-alloyed steel. Strak et al. [54] patented a method for inhibition by treating the unit with group of acids containing dodecenyl succinic acid and di hexyl succinic acid. Leinweber et al. [55] patented a water soluble biodegradable inhibitor of metal salt of CH_3_SCH_2_CH_2_CH(NHCOR)COOH containing anionic and cationic surfactant. Abdrakhmanov et al. [56] studied N-acetyl-2-(2,3-dihydroxy cilopentenyl) aniline with concentration 50–200 mg/L as inhibitor for water petroleum solution including H_2_S. The inhibitive effect of natural honey on corrosion of petroleum pipelines and high salinity are studied. El-Etre et al. [57] studied the effect of fungi on corrosion inhibition efficiency and found that it is decreased in presence of natural honey in high salinity water. It efficiently works in presence of dissolved iron cation. Li et al. [58] tested fatty acid and quaternary ammonium salt as a corrosion inhibitor which, reduces oil-water interfacial tension in very low concentrations as a result steel surface becomes hydrophobic. The performance of the inhibitor can be enhanced when steel surface is periodically wetted with oil phase [58].

## 3. Inhibitor Used in Boiler Tubes

**Figure 2:**
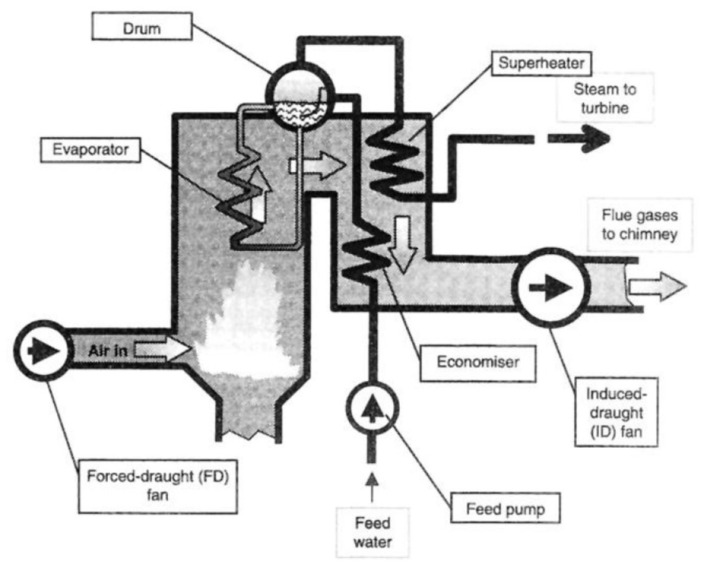
Schematic of a boiler [59].

The boiler tube is being used in power generation, chemical industries and oil refinery where heat water flow as shown in Figure 2 [59]. It is susceptible to corrosion owing to the high temperature and composition of exhaust gas. In the boiler, the exhaust gas is transferred to the evaporator tubes from the steam/water circuit where steam leaves the drum, enters a bank tubes and significant amount of heat is adsorbed from the gases present in steam/water. There are many components of super heater suspended in the flue gas which, contain many aggressive ions where they react with components of boiler tubes and started the corrosion. Thus, it requires an inhibitor to reduce the corrosion of suspended component of boiler tubes. The solubility of the silica, iron, manganese, organics, oxygen, etc., is lower in water where they get adsorbed onto the boiler components and causes scaling as well as corrosion [60]. However, the scaling can be controlled by water softening equipment, pH adjustment, chemical doses and physical water treatment method. Chemicals used for scale treatment are polymers (polyacrylate, etc.), polymethacrylate, phosphonate, sodium phosphates (NaH_2_PO_4_, Na_2_HPO_4_, Na_3_PO_4_, NaPO_3_), chelants (EDTA, NTA), co-polymers and polyphosphates, which control the calcium scale formation. Most of the scales or deposits are non-metallic, which possess low thermal and electrical conductivity resulting in a reduction in heat transfer and efficiency of the boiler. Due to the reduction in thermal conductivity, the tube wall temperature would increase which, causes rupture in components as well as decrease the efficiency [61]. The scale deposits onto the boiler components are different oxides, which leads to increase the wall temperature and hinder the proper flow of water. Corrosion is the next most important problem in boiler tubes where Fe^++^ comes in running water and dissolve, thus, the thickness of boiler components tends to decrease. In the meantime, O_2_ is reduced to hydroxyl (OH^−^) or hydrogen evolution reaction at cathodic side [60].

The electricity generated by thermal power plants is from the burning of coal, which boil water resulting production of steam [62]. Most of the coal contains a high content of sulfur, non-combustible ash and abrasive minerals, which causes erosion corrosion of boiler tubes [63,64]. Such a type of corrosion problems occurs in power generation equipment, turbine used in ships, aircraft, energy conversion and chemical industries. However, the corrosion mitigation can be achieved using super alloys, corrosion inhibitors and coatings [65].

There are different types of corrosion occur in the boiler tubes. These are uniform, pitting and galvanic corrosion. Therefore, it is necessary to remove/descale the corrosion products from the metallic components. The descaling can be carried out by chemical where the composition of chemical is very important to remove the oxides from the metal surfaces. It depends on scale formation. The chemical cleaning is selective where a particular chemical removes a specific types of scale. The most of the chemicals being used in descaling are inhibited inorganic (HF, HCl, H_2_SO_4_ and HNO_3_) and organic (citric acid, glycolic acid, formic acid, etc.) acids. The silicate deposits can be only removed by HF whereas oily and fatty acid by tri-sodium phosphate, caustic soda or boiled alkaline solutions. Moreover, the effective chemicals to remove the scale are HCl and H_2_SO_4_. Since, there is difference in effectiveness of these acids. Among them, the most effective and popular acid is HCl followed by H_2_SO_4_. From the experimental studies it is concluded that inorganic acid is more efficient than organic acid. A 5% HCl solution is efficient for effective cleaning of scale deposits. However, if the concentration of HCl is increased, it causes the corrosion attributed to the dissolution of metal in acid solution [66] as described in Equations (5–8). The Cl^−^ ions in higher concentration of HCl react with metals and form FeCl_2_/FeCl_3_ at higher temperature and combined with O_2_ to form oxides as well as liberate chlorine [67]. Thus, it can be said that high concentration of HCl in boiler tube (where temperature is more than 500 °C) catalyze the corrosion reaction resulting severe corrosion. This oxide film is porous, which work as reservoir for oxygen, moisture and other aggressive ions and intensify the corrosion reaction [67]. Therefore, it is suggested that at high temperature, the corrosion of metal components is significantly increased in high concentration of HCl rather than reduction.

There is a huge amount of economic and resource loss around the world to combat the corrosion of boiler tubes. Understanding the causes of cracking and corrosion enable us to develop strategies that reduce corrosion rate and inhibit cracking. Therefore, inhibitors are being used worldwide to mitigate or reduce the corrosion of boiler tubes. The amine is considered as organic [68], whereas phosphate is in inorganic inhibitors [69] which is the most effective inhibitor, due to reducing the corrosion of boiler tube significantly.

## 4. Inhibitors Used to Control the Dissolution of Metal Components in Oil-Well

Inhibitors are being used to combat the corrosion of steel of oil-well in different acid solutions. The details about the inhibitors used in different acid solutions are described below.

### 4.1. In Hydrochloric Acid (HCl)

HCl is used to remove the oxide film deposit onto the steel surface in oil-well industries. The concentration of HCl can vary between 5% to 35%. It dissolves black oxide films, limestone, dolomite and other impurities from the surface. Many additives such as surfactant, alcohol, suspending agents, sequestering agent, anti-sludge agents and inhibitors are added to solve the problems created in oil-well such as emulsion, corrosion and sludge.

Organic and inorganic acids are used for acidizing in oil wells, but due to the aggressive in nature, they cause corrosion of tubes. O_2_ is introduced in well during drilling process while CO_2_, H_2_S and water injected for secondary recovery. These three are aggressive gases (O_2_, CO_2_ and H_2_S) and complicate the problem of inhibitor in oil-well. The choice of inhibitors used in oil and gas field depends on area such as refineries, pipelines, wells and recovery units. Corrosion in petroleum refineries is due to naphthenic acid and in crude oil pipelines due to S compound. Oil and water-soluble inhibitors are used in pipelines, while slag are used to stop the formation of deposits. Both inorganic and organic inhibitors can be used for oil-well treatment, but inorganic inhibitors cause environmental problems therefore, organic inhibitors are employs specially those containing N, S and O heterocyclic compounds, quaternary ammonium salt and acetylenic compounds in acidic medium.

**Figure 3:**
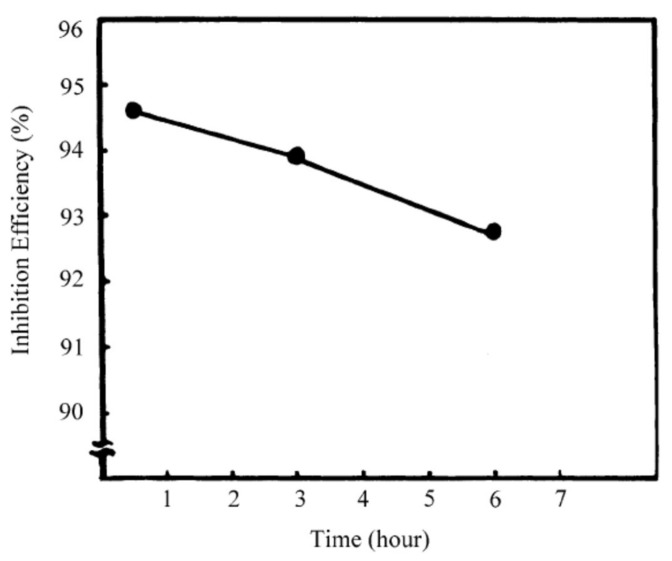
Variation of inhibition efficiency with immersion time of UMOD for N-80 steel in 15% boiling HCl from weight loss measurements [70].

**Figure 4:**
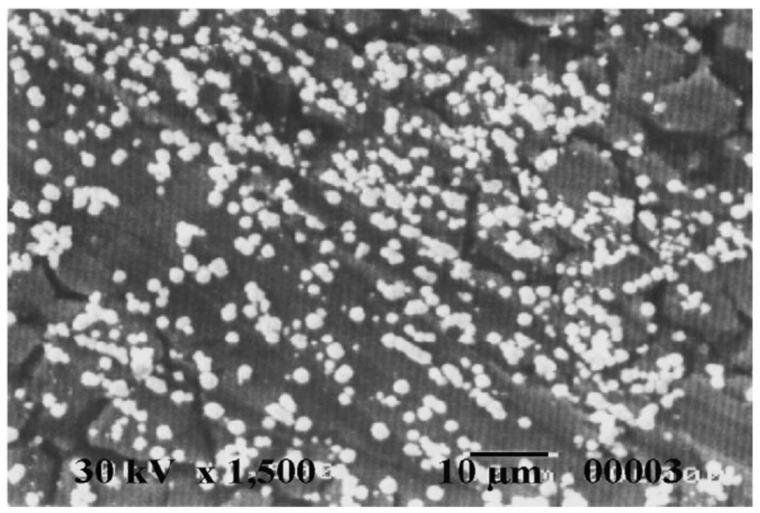
Micrographs of mild steel after immersion in 2 M HCl solution in the presence of 400 ppm of the inhibitor [72].

**Figure 5:**
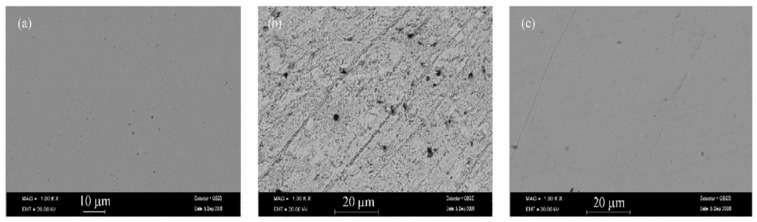
SEM of mild steel (**a**) after polishing, (**b**) after immersion in 1.0 mol·L^−1^ HCl and (**c**) after immersion in 1.0 mol·L^−1^ HCl + 0.04% Na-CMC [75].

Quraishi and Jama [70] have synthesized eco-friendly fatty acid as corrosion inhibitor for mild steel in 15% HCl at 105 ± 2 °C and studied their effect by weight loss method. They have found that 2-undecane-5-mercapto-1-oxa-3,4-diazole (UMOD) is the best inhibitor and exhibited 94% efficiency. These compounds contain lone pair of electrons on N and S atoms and π electrons in heterocyclic ring through which they can adsorb on metal surface. They work as mixed type of inhibitor and effective for corrosion of mild steel in 15% boiling HCl. The efficiency of inhibitor is decreased with increase in duration as shown in Figure 3. Mohammed-Dabo et al. [71] have studied the inhibiting effect of Ficus abutilifolia (FA) extract on N-80 steel in 15% HCl by weight loss techniques and they have found that the maximum efficiency was obtained at the optimum concentration. However, once the temperature is increased, the efficiency is decreased. Moreover, they have compared the efficiency of FA with well-known corrosion inhibitor i.e., propargyl alcohol (PA). They have found that the maximum efficiency can be obtained by 59.12% at 30 °C while PA exhibited 96.79%. The inhibition efficiency of both inhibitors are increased with increase in concentration while decreased with increase in temperature. Migahed et al. [72] synthesized corrosion inhibitor for mild steel in 2 M HCl solution from recycled polyethylene terephthalate (PET). PET was depolarized by triethanolamine into glycolyzed product (GT) followed by bromacetic acid in presence of manganese acetate as a catalyst to obtain GT-Br. The obtained product was reacted with thiourea to give thiol derivatives (GT-SH). Its effectiveness was studied and found that efficiency is increased with increase in concentration. SEM observations of electrode surface showed a film of inhibitor molecule (Figure 4). This inhibitor retarded both cathodic and anodic reactions of steel surface. Ali et al. [73] have synthesized new compounds as corrosion inhibitors from cycloaddition of cyclic nitrone-1-pyrroline-1-oxide with many alkenes, phenyl isocynate and phenyl isothiocynate. The synthesized inhibitors showed excellent inhibition efficiency for mild steel in HCl attributed to the presence of heteroatoms (N and O). Khaled [74] studied inhibitive effect of some benzimidazole derivatives i.e., 2-aminobenzimidazole (AB), 2-(2-pyridyl) benzimidazole (PB), 2-aminomethylbenzimidazole (MB), 2-hydroxybenzimidazole (HB) and benzimidazole (B) on corrosion inhibition of mild steel in 1 M HCl solution. The inhibition efficiency is found to be AB > PB > MB > HB > B. The AB exhibited highest efficiency owing to the presence of amino group, which enhanced the adsorption by increasing the availability of π electrons of the ring. The sodium carboxymethyl cellulose (Na-CMC) works as a mixed type of inhibitor in 1.0 mol L^−1^ HCl by forming a uniform and dense protective layer on mild steel surface [75] as shown in Figure 5.

CO_2_ corrosion inhibition of N80 steel in liquid single-phase and liquid/particle two-phase flow by 2-undecyl-1-hydroxyethyl imidazoline (HEI-11) and 2-undecyl-1-hydroxyethyl-1-hydroxyethyl quaternary imidazoline (HQI-11) was investigated by Liu et al. [76]. The results show that the corrosion rate in the absence and presence of the imidazoline is strongly dependent on the flow condition and presence of entrained sand particles. Amosa et al. [77] evaluated some selected environmentally benign iron compounds (synthetic magnetite and ferrous gluconate) as corrosion inhibitors for oil-well steel (N80) in 50 mg/L sulfide at various pH ranging from 5.5 to 11.5 as well as elevated temperature and pressure. The ferrous complex is found to be a better corrosion inhibitor compared to the synthetic magnetite. It exhibited up to 99.2% inhibition efficiency (IE) when the dose of the scavenger was doubled (i.e., when the sulfide to scavenger ratio was 1:2) irrespective of other factors such as pH, temperature and pressure. Whereas, the synthetic magnetite exhibited maximum 75.1% efficiency at 1:4 sulfides to scavenger ratio on the lowest pH i.e., pH 5.5, which is not desirable for a drilling mud. Therefore, there is requirement of green inhibitor for acid solution. The updates on the ecofriendly corrosion inhibitor used in HCl is described in Table 1. From this table, it can be seen that green inhibitor in HCl solution exhibited excellent performance in corrosion reduction of steel.

The nitrite-based inhibitor has been used in simulated cooling water to reduce the corrosion in neutral and alkaline condition, however, it enhances the corrosion in acidic solution. Siragul et al. [78] have used sodium nitrite to reduce the corrosion of mild steel and they have found that 500 ppm shown the excellent corrosion resistance at pH 8 attributed to the formation of Fe_2_O_3_ onto the surface. The amino acids i.e., glycine, valine and leucine which, has longer hydrocarbon chain exhibited good corrosion inhibition for 316 L stainless steel in 1 M H_2_SO_4_ solution [79]. Among them, glycine has the longest hydrocarbon chain, thus, it shows the highest inhibition efficiency i.e., 84.2%. The organic based inhibitors containing hetero atoms are performing excellent in H_2_SO_4_ and HCl owing to the chemisorption [80]. H_2_SO_4_ is used in oil well drilling and scale removing in boiler. Thus, in the subsequent paragraph, the inhibitor being used to reduce the corrosion of steel in H_2_SO_4_ solution is discussed below.

### 4.2. In Sulphuric Acid (H_2_SO_4_)

**Figure 6:**
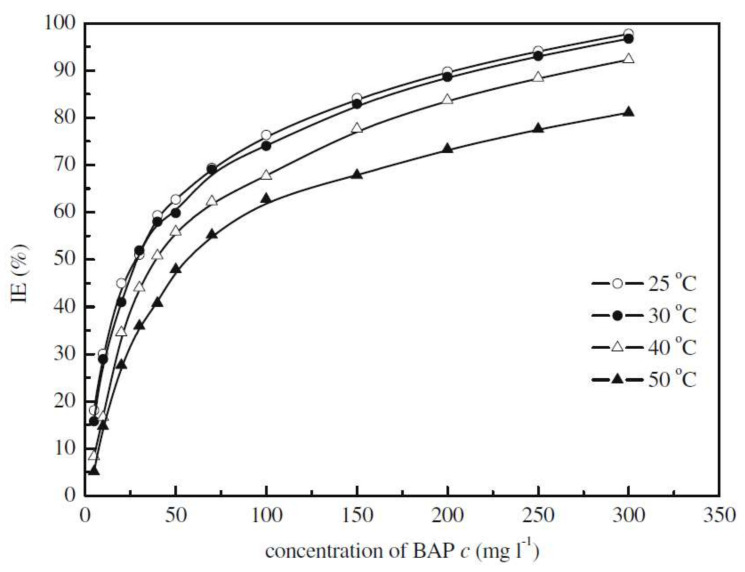
Relationship between inhibition efficiency (IE) and BAP concentration in 1.0 M H_2_SO_4_ [76].

**Figure 7:**
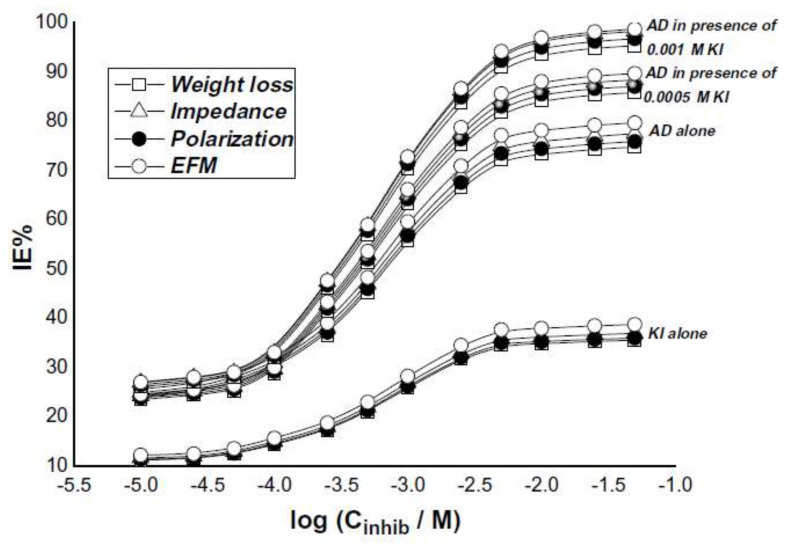
Dependence of the inhibition efficiency values (IE%) obtained from weight loss, polarization, impedance and EFM methods recorded for a LCS in 4.0 M H_2_SO_4_ solutions containing different concentrations of KI alone, AD alone and various concentrations of AD in presence of 0.0005 and 0.001 M KI [81].

Acid solutions are very aggressive to remove the oxides formed onto the metallic substrate. However, while using acid solutions, they cause metal loss, thus, it is required to add some inhibitors which, reduce the dissolution of metals and alloys. The hetero atoms i.e., N, S and O in chemical formula are effective to reduce the metallic dissolution in acid solution. 6-benzylaminopurine (BAP) has worked effectively to reduce the corrosion of cold rolled steel in H_2_SO_4_ solution and found that its inhibition efficiency increases with concentration but decrease with increase in temperature [76] as shown in Figure 6. BAP possess its inhibition by forming BAP-Fe^2+^ complex as a protective film on steel surface in H_2_SO_4_ solution. Rehim et al. [81] have studied the influence of adenine (AD) as a green inhibitor on the corrosion resistance performance of low carbon steel (LCS) in aerated 4.0 M H_2_SO_4_ solutions using different techniques such as weight loss, potentiodynamic polarization, electrochemical impedance spectroscopy and electrochemical frequency modulation (EFM). They have found that the inhibition efficiency has increased with increase in AD concentration in presence of KI (synergism) and immersion time as shown in Figure 7.

**Figure 8:**
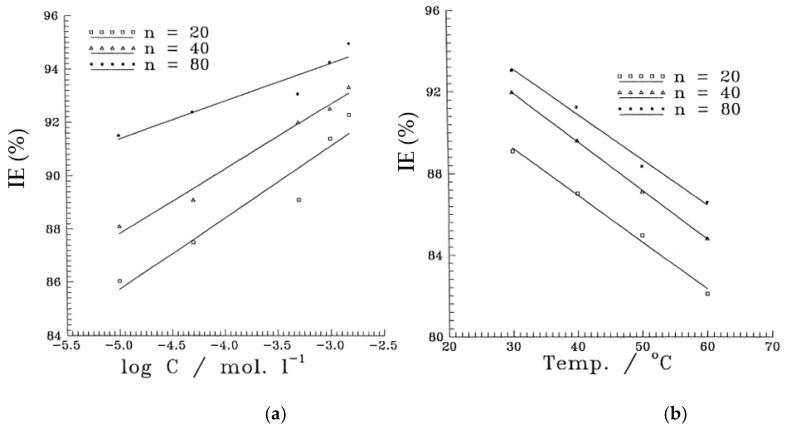
Variation of the protection efficiency with (**a**) the logarithmic concentrations of the inhibitors in 1.0 M H_2_SO_4_ at 30 °C and (**b**) temperatures at concentration 5 × 10^−4^ M inhibitors [82].

**Figure 9:**
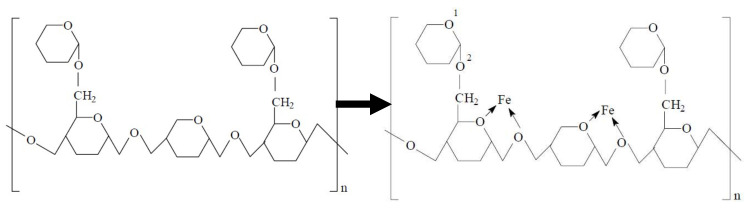
Corrosion protection mechanism of Guar gum in 1 M H_2_SO_4_ solution [83].

Fatty acid is considered as eco-friendly corrosion inhibitor in aggressive solution. E.E.Foad [82] have studied the inhibition efficiency of some ethoxylated fatty acids, i.e., OL[EO]20, OL[EO]40 and OL[EO]80, and they have found that all fatty acids exhibited more than 80% efficiency as well as once the concentration is increased, efficiency is increased (Figure 8a) even though at 60 °C (Figure 8b) owing to the adsorption of inhibitor molecules onto the substrate. Polysaccharide is a naturally occurring organic compounds which, shows excellent corrosion inhibition in acidic solution. M. Abdallah [83] has used Guar gum (a polysaccharides consisting straight chain of D-mannopyranose) as a corrosion inhibitor for carbon steel in 1 M H_2_SO_4_ solution using weight loss and Tafel polarization techniques. The corrosion inhibition of Guar gum on carbon steel is due to adsorption at electrode/solution interface as shown in Figure 9 where repeated pyrane moiety (lone pair of electrons of O) in polysaccharide compound adsorbed onto the steel substrate (Fe) by forming a chelate. Zizyphus Spina-Christi (ZSC) extracts acts as corrosion inhibitor for steel in 1.0 M H_2_SO_4_ containing 10% ethyl alcohol solution and obtained more than 90% efficiency accessed by different techniques [84]. ZSC is a mixed type of inhibitor which, inhibit the corrosion reactions at cathodic and anodic sites of steel. Tryptamine (TA), a derivative of the tryptophan is established to be an effective corrosion inhibitor for ARMCO iron in 0.5 M deaerated H_2_SO_4_, which adsorbed onto the steel by forming a complexes between ferrous ions adsorbed on iron surface (Fe^++^_(ads)_) and TA molecules [85]. TA exhibited 90–99% inhibition efficiency at 25–55 °C over time. The effect of cysteine (Cys, a natural occurring amino acid) on corrosion inhibition of steel in H_2_SO_4_ solution is studied by Özcan et al. [86] and they have described that cysteine molecules adsorbed at the metal/solution interface and exhibited excellent corrosion inhibition.

The cationic surfactant i.e., cetyl trimethyl ammonium bromide (CTAB), which used in isolation of high molecular weight DNA in plants. Maghraby and Soror [38] have used CTAB as corrosion inhibitor for carbon steel in different concentration of H_2_SO_4_ and they have found that CTAB has adsorbed onto the steel surface by Van der Waals force. The corrosion protection of CTAB is given by chemisorption mechanism at steel/solution interface. The thiazole-based inhibitor has established an excellent corrosion inhibitor which, act as mixed type of inhibitor. It blocks the cathodic and anodic sites of steel and adsorbed onto the steel substrate in 1 M H_2_SO_4_ solution [87]. Polyvinyl pyrrolidone (PVP) is itself a good corrosion inhibitor but once iodide ions were added in PVP, it increases the corrosion resistance properties of steel substrate attributed to the synergistic effect of iodide ion with PVP in acid condition [88].

The eco-friendly corrosion inhibitor exhibited more than 99% efficiency (Table 1) in mitigation of steel, which is economic, less hazardous and easy to application. Alternatively, the other organic inhibitors are also exhibited more than 90% efficiency as discussed above but they are expensive, hazardous and need proper attention in synthesis. Thus, it is suggested to use green inhibitor to combat the corrosion of steel structures. 

**Table 1:** Latest updates from 2019 to till date on eco-friendly inhibitor to mitigate the corrosion of steel in acidic environment.

Sl. No.	Name	Medium	Application	Efficiency (%)	Ref.
1	Agaricus bisporus	HCl	mild steel	98.9	[89]
2	2-(5-(4-cyanophenyl) -2,4,6,8-tetraoxo-1,2,3,4,6,7,8,9—octahydropyrido [2,3-d:6,5-d’] dipyrimidin-10 (5 H)-yl)-3-(1H-imidazol-4-yl) propanoic acid (CTDP)	HCl	mild steel	94.01	[90]
3	Stevioside –Zn^2+^	HCl	carbon steel	95.68	[91]
4	Polygonatum cyrtonema Hua	HCl	mild steel	94.73	[92]
5	4-(2,4,6,8-tetraoxo-2,3,4,5,6,7,8,9-octahydro-1H-pyrano [2,3-d:6,5-d’]dipyrimidin-5-yl)benzonitrile (OPDB)	HCl	mild steel	91.11	[90]
6	chitosan derivatives CS-1	HCl	mild steel	93.5	[93]
	chitosan derivatives CS-2	HCl	mild steel	97.3	
7	Purine derivatives N-benzoylaminopurine (N-BAP)	HCl	mild steel	98.6	[94]
	Purine derivatives6-furfurylaminopurine (FAP)	HCl	mild steel	90.5	
8	imidazole ionic liquids and citric acid-based carbon dots	HCl	Carbon steel	92.6	[95]
9	(E)-4-(2-(4-fluorobenzylidene)hydrazinecarbonyl)-1-propylpyridin-1-ium iodide (Ipyr-C3H7)	HCl	mild steel	90.1	[96]
10	Aloe polysaccharide	HCl	mild steel	96.12	[97]
11	silver nanoparticles: (SNPs)-chitosan (CT) nanocomposite	HCl	mild steel	97.5	[98]
12	4-aminoazobenzene modified natural glucomannan	HCl	mild steel	95.2	[99]
13	Aminoantipyrine derivatives	HCl	P110 steel	92.1	[100]
14	Epoxy sugar based glucose derivatives	HCl	carbon steel	96.8	[101]
15	Dardagan Fruit extract	HCl	mild steel	92	[102]
16	Biowaste [human hair-(HHR)]	HCl	mild steel	96.65	[103]
17	Pachysandra terminalis	HCl	carbon steel	95.79	[104]
18	Gentiana olivieri	HCl	mild steel	93.70	[105]
19	Petroselium sativum	HCl	mild steel	92.39	[106]
20	Betel leaves	HCl	Q235 steel	95.4	[107]
21	Polar group substituted imidazolium zwitterions	HCl	mild steel	94.59	[108]
22	Cashew nut shell liquid (CNSL)	HCl	carbon steel	99.47	[109]
23	Ceratonia Siliqua L seeds	HCl	carbon steel	95	[110]
24	Meat extract inhibitor	HCl	mild steel	94.8	[111]
25	Quinoa seed	HCl	carbon steel	98	[112]
26	Anisole	HCl	mild steel	86	[113]
27	Natural nutmeg oil	HCl	carbon steel	94.73	[114]
28	Hymenaea stigonocarpa	H_2_SO_4_	mild steel	90	[115]
29	imidazole ionic liquids 1,4-bis(3-methylimidazolium-1-yl)butane dibromide	H_2_SO_4_	mild steel	92.9	[116]
	imidazole ionic liquids 1-aminopropyl-3-methylimidazolium bromide ([APMIm]Br)	H_2_SO_4_	mild steel	88.0	
	imidazole ionic liquids 1-propyl-3-methylimidazolium bromide ([PrMIm]Br)	H_2_SO_4_	mild steel	69.6	
30	3-(4-fluorobenzyl) -1-methyl-1H-imidazol-3-ium bromide	H_2_SO_4_	mild steel	99.48	[117]
31	Rhododendron schlippenbachii	H_2_SO_4_	carbon steel	92.24	[118]
32	Lavandula and Ricinus Communis Oil	H_2_SO_4_	mild steel	96.35	[119]
33	Chicken nail extracts	H_2_SO_4_	mild steel	74.04	[120]
34	Rosuvastatin drug	H_2_SO_4_	mild steel	92	[121]
35	Aminothiazolyl coumarin	H_2_SO_4_	mild steel	71.36	[122]
36	Litchi Chinensis	H_2_SO_4_	mild steel	97.8	[123]
37	Leech extract	H_2_SO_4_	carbon steel	91	[124]
38	Cyclohexylamine	H_2_SO_4_	mild steel	81.06	[125]
39	Clove essential oil extract with basil and atlas cedar oil	H_2_SO_4_	mild steel	90	[126]
40	Macaranga peltata leaves	H_2_SO_4_	mild steel	92.6	[127]
41	Citrus sinensis essential oil	H_2_SO_4_	carbon steel	76.95	[128]

### 4.3. In Organic Acid Solution

There are a limited number of studies carried out by researchers worldwide on organic acid solution to inhibit the dissolution of metals and alloys compared to mineral acids [129,130,131,132]. Although the organic acids are weak acid and easily dissociate, which cause corrosion or dissolute the metals. However, the corrosion caused by aqueous organic acids/solvents can effectively control the corrosion if used with inhibitors [133]. The use of organic inhibitors (HCOO^−^) reduce the corrosion of metals and alloys by forming a passive film as described below [134].

Fe +HCOO^−^ → [Fe(HCOOH)]_ads_ + e^−^ (9)

[Fe(HCOOH)_ads_ → Fe(HCOOH)^+^ + e^−^ (10)

(11) $$[\mathrm{Fe}(\mathrm{HCOOH}){]}^{+}\;+\;H^{+}\;↔\;{\mathrm{Fe}}^{++}\;\mathrm{HCOOH}$$

**Figure 10:**
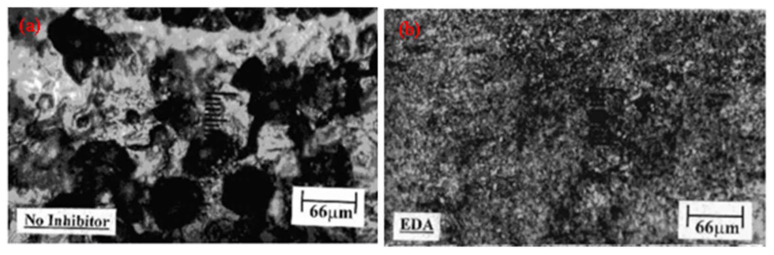
Optical micrographs showing (**a**) pitting (without inhibitor) and (**b**) adsorbed layer by 4% EDA on carbon steel surface in 16% petroleum water [135].

Amine is an effective corrosion inhibitor to reduce the dissolution of steel. Amine has nitrogen atom with a lone pair of electrons in its molecular structure, which effectively adsorb onto the steel surface by donating its lone pair of electrons to vacant d-orbital of Fe. Ashassi-Sorkhabi et al. [135] have studied the effect of different amines on the corrosion of carbon steel in petroleum water containing acetic acid and NaCl and they have found that ethylenediamine (EDA) is the most effective inhibitor to reduce the corrosion owing to the formation of adsorbed FeOH_ads_ and FeX_ads_ which, lower the anodic reaction compared to without inhibitor, as seen in optical micrographs (Figure 10).

The heteroatoms, i.e., N, O and S, in cyclic aromatic and non-aromatic rings adsorbed onto the surface and reduce the corrosion of steel in organic acid solution. The thiourea derivatives i.e., phenyl thiourea (PTU), tolyl thiourea (TTU), diphenyl thiourea (DPTU) and ditolyl thioure (DTTU) acted as corrosion inhibitors for mild steel in 20% formic acid. Their corrosion inhibition is explained on the basis of their molecular structure and order of inhibition efficiency is as follows: DTTU > DPTU > TTU > PTU [136]. The result showed that all compound behaved as mixed type inhibitors and their inhibition property is owing to adsorption of inhibitor molecule on metal surface and obey Temkin’s adsorption isotherm. Different fatty acid triazoles i.e., 5-Heptadec-8-enyl-4-phenyl-4H–[1,2,4] triazole-3-thiol (HPTT), 4-phenyl-5-undecyl-4H–[1,2,4] triazole-3-thiol (PUTT) and 5-dec-9-enyl-4-phenyl-4H–[1,2,4] triazole-3-thiol (DPTT) were synthesized, and their corrosion resistance performance for mild steel was accessed in 20% formic acid. The corrosion inhibition efficiency of these compounds varies with immersion time, concentration and temperature. However, all inhibitor exhibited more than 90% efficiency and work as mixed inhibitor even at lower concentration i.e., 25 ppm in acid solution [137].

**Figure 11:**
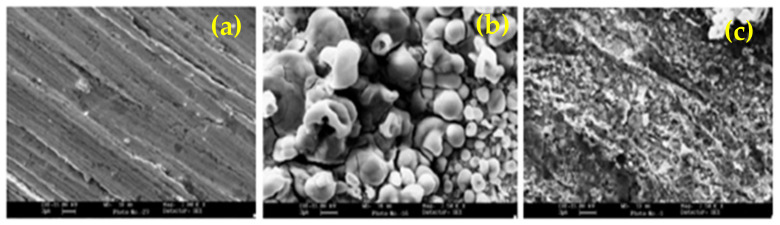
Scanning electron micrographs for: (**a**) polished mild steel, (**b**) mild steel in 20% formic acid and (**c**) mild steel in 20% formic acid +100 ppm APT [139].

The thiazole derivatives i.e., 2(N, N dimethylamino) benzylidene imino-4-(4-methyl phenyl)-1,3-thiazole (DIMPT), 2-benzylidene- imino-4-(4- methyl phenyl)-1,3-thiazole (BIMPT), 2-salicylidene imino-4-(4-methylphenyl)-1,3-thiazole (SIMPT) and 2-cinnamylidene imino-4-(4-methyl phenyl)-1,3-thiazole (CIMPT) were synthesized in the laboratory and their influence on the corrosion inhibition of mild steel in 20% formic acid and 20% acetic acid was investigated. The inhibition efficiency of these compounds was found to vary with their nature and concentration, temperature, immersion time and acid concentration. The adsorption of all the thiazoles on mild steel surface was found to follow the Langmuir adsorption isotherm as well as retard the cathodic and anodic dissolution [138]. Alternatively, Rafiquee et al. [139] have synthesized 2-amino-1,3,4- thiadiazoles (AT), 2-amino-5-methyl—1,3,4- thiadiazole (AMT), 2-amino-5-ethyl-1,3,4- thiadiazoles (AET) and 2-amino-5-propyl- 1,3,4- thiadiazoles (APT) and accessed their corrosion inhibition in 20% formic acid and 20% acetic acid. They have found that 100 ppm APT exhibited highest efficiency as well as adsorbed onto the steel surface and exhibited uniform passive film (Figure 11).

## 5. Conclusions and Recommendations

The corrosion of structural steel is severe problem of the world. Therefore, it needs utmost attention of the researchers to reduce corrosion issues of the steel, which lead to control the economy loss. There are different processes being adopted by the researchers to combat the corrosion of steel, including development of high strength, low alloys, corrosion resistant steel as well as different coatings and inhibitors. In present state-of-art review articles, I have demonstrated about the inhibitor being used to reduce the corrosion of steel in descaling, acid pickling, pipeline, boiler tube and oil-well using organic and eco-friendly corrosion inhibitors. It is well known that most of the organic inhibitor is effective attributed to the having hetero atoms, i.e., O, S and N, in chemical formula, which adsorbed onto the steel surface and leads to provide protection against corrosion in acid solution and cease the cathodic as well as anodic reaction at steel/acid interface. However, they are expensive, hazardous, and needs expert in synthesis. Alternatively, green corrosion inhibitor is economical, eco-friendly and can be procured from the natural resources. There is more attention required by the researchers to develop and study on green inhibitor used in HCl and H_2_SO_4_ solution, which exhibited more than 90% inhibition efficiency. Thus, it is suggested to expand the research and make attention on eco-friendly corrosion inhibitor for sustainable society.

## Data Availability

Not applicable.
